# Temporal Associations and Heterogeneity of Diagnosis-Related Group (DRG) Payment Reform with Hospitalization Costs Among Patients with Colorectal Cancer in China

**DOI:** 10.3390/healthcare14182988

**Published:** 2026-09-12

**Authors:** Zhiyi Luo, Biao Fan, Hongyuan Wu, Shenqi Han, Zihao Bian, Ning Zhao, Zongjiu Zhang, Shuyuan Cheng

**Affiliations:** 1 Institute for Hospital Management, Tsinghua University, Beijing 100084, China; luozy24@mails.tsinghua.edu.cn (Z.L.); drfanbiao@bjmu.edu.cn (B.F.); wu-hy25@mails.tsinghua.edu.cn (H.W.); hansq25@mails.tsinghua.edu.cn (S.H.); bianzh24@mails.tsinghua.edu.cn (Z.B.); zhaoningts@tsinghua.edu.cn (N.Z.); 2 Key Laboratory of Carcinogenesis and Translational Research (Ministry of Education/Beijing), Gastrointestinal Cancer Center, Peking University Cancer Hospital & Institute, Beijing 100142, China; 3 School of Medicine, Tsinghua University, Beijing 100084, China; 4 The First Affiliated Hospital of Sun Yat-sen University, Guangzhou 510080, China

**Keywords:** Diagnosis-Related Groups (DRGs), colorectal cancer, hospitalization cost, interrupted time-series analysis, heterogeneity analysis

## Abstract

**Background/Objectives**: We aimed to evaluate the temporal associations and heterogeneous patterns between the Beijing Diagnosis-Related Group (DRG) 2.0 payment reform and hospitalization costs and resource utilization among patients receiving major colorectal cancer (CRC) surgery and to explore hospital adaptive cost adjustment behaviors under bundled payment constraints. **Methods**: Utilizing inpatient data of 1232 colorectal cancer surgical patients from a Beijing hospital spanning January 2021 to October 2024, we adopted segmented regression interrupted time-series analysis (ITSA), with April 2022 defined as the policy intervention point. The analysis used total hospitalization expenses, itemized costs, cost composition proportions, and length of stay (LOS) as outcome indicators, conducted heterogeneity analysis, and applied seasonal autoregressive integrated moving average (SARIMA) counterfactual forecasting as a supplementary sensitivity check. All medical expenditures were inflation-adjusted based on Beijing’s medical consumer price index (CPI), with 2024 as the base year. **Results**: After DRG implementation, total hospitalization costs showed an immediate decrease of 13,111.73 CNY and a sustained monthly downward trend of 1312.60 CNY. Medical consumable fees were the main component associated with total-cost reduction, and their proportion declined immediately by 4.1 percentage points. LOS showed no abrupt immediate decline but shortened by 0.22 days per month over the post-reform period. Heterogeneous association patterns were observed across selected clinical and treatment subgroups. SARIMA counterfactual forecasting provided supplementary, directional sensitivity evidence for the primary outcomes and was interpreted cautiously for volatile sub-item expenditures. **Conclusions**: DRG 2.0 reform was associated with lower hospitalization costs and improved bed-turnover efficiency among CRC surgical patients, mainly through reductions in consumable expenditures.

## 1. Introduction

Colorectal cancer (CRC) is one of the most common and high-burden malignancies worldwide. In China, approximately 517,000 new CRC cases and 240,000 CRC-related deaths occurred in 2022, with CRC ranking second among all malignant tumors in terms of incidence, while its mortality burden also remained among the highest [1]. With population aging, changes in lifestyle, and improvements in screening, diagnosis, and treatment, the demand for CRC-related medical services has continued to increase, making CRC an important disease burden affecting population health and the sustainable operation of medical insurance funds [2].

CRC risk and related hospitalization costs are shaped by interacting demographic, behavioral, clinical, and environmental determinants. Aging, dietary transition, obesity, physical inactivity, smoking, alcohol consumption, family history, inflammatory bowel disease, delayed screening, and tumor-stage differences can all influence disease occurrence, treatment complexity, postoperative recovery, and cost intensity. Broader social and environmental changes may also affect health burden and medical expenditure: for example, pollution has been discussed as a potential health-cost driver, and urbanization, income growth, energy use, and pollution have been linked to population health pressures [3,4]. Therefore, CRC surgery is an appropriate tracer condition for evaluating payment reform because it combines a high and growing disease burden, relatively standardized surgical pathways, expensive consumables and medications, and substantial heterogeneity in clinical severity and resource needs.

Reforms of China’s medical insurance payment system have continued to advance in recent years. Diagnosis-related group (DRG)-based payment changes the incentive structure under the traditional fee-for-service model. Furthermore, each DRG is assigned a fixed pre-set reimbursement threshold calculated based on historical average resource consumption of homogeneous cases within the same group. Hospitals bear the financial deficit if the actual total hospitalization cost of a patient case surpasses this fixed threshold. This payment threshold creates financial pressure for hospitals and is important for evaluating provider adaptive cost-containment behaviors and the rationality of DRG tariff setting. In March 2022, the Beijing Municipal Medical Insurance Bureau prioritized the implementation of DRG payment and established and improved a DRG payment and performance management system.

Existing studies on DRG payment have provided important evidence regarding total costs, average length of stay, and healthcare service efficiency. Some studies have shown that DRG payment can generally reduce average inpatient costs and length of stay [5,6] while also indicating potential risks such as reductions in service quality and patient selection [7]. In the field of oncology, DRG reform may improve the homogeneity of treatment costs for certain malignant tumors, although differences exist across treatment modalities [8]. However, a critical review of the literature indicates several remaining gaps. First, many evaluations are conducted at the aggregate hospital level, regional level, or across broad disease categories, so evidence for oncology-specific and single-disease surgical populations remains insufficient. Second, the total hospitalization cost and LOS are commonly reported, but fewer studies decompose cost structures to determine whether cost containment is associated with consumables, medications, diagnostic services, treatment services, or service fees. Third, heterogeneity is often described through separate subgroup estimates, while formal interaction evidence is less frequently reported. Fourth, concurrent influences, such as pandemic-related disruptions, changes in surgical approach, procurement policies, and clinical pathway management, may coexist with DRG reform and complicate interpretation. These gaps leave unclear how DRG reform is temporally associated with CRC surgical hospitalization costs, which cost components are most closely linked to reform-period changes, and which patient or treatment subgroups show different association patterns.

Accordingly, the research questions were explicitly defined as follows: (1) Was the Beijing DRG 2.0 payment reform associated with immediate and sustained changes in total hospitalization costs among patients undergoing major CRC surgery? (2) Which cost components and cost proportions contributed most to any observed cost changes? (3) Was DRG reform associated with changes in resource utilization, measured by LOS? (4) Did the policy associations differ across patient baseline characteristics, surgical and antitumor-treatment modalities, and insurance-related subgroups after formal interaction testing? (5) Were the main findings robust after considering COVID-19-related disruptions, monthly admission volume, surgical approach imbalance, and counterfactual SARIMA sensitivity analysis?

The innovations of this study are mainly reflected in three aspects. First, the study population is focused on patients with CRC undergoing major surgery, addressing the insufficiency of oncology-specific and single-disease studies in existing DRG research. Second, this study not only examines total hospitalization costs and length of stay but also further analyzes changes in cost structures, including medication fees, consumable fees, diagnostic fees, treatment fees, and service fees, thereby helping to identify the cost components most closely associated with DRG reform. Third, this study conducts heterogeneity analyses across three dimensions, patient baseline characteristics, surgical treatment characteristics, and medical insurance characteristics, which may provide empirical evidence for optimizing oncology-related DRG payment standards, special-disease case-by-case review management, and precision medical insurance regulation.

## 2. Materials and Methods

### 2.1. Study Design

Interrupted time-series analysis (ITSA) is a quasi-experimental design used to evaluate population-level policy interventions implemented at a clearly defined time point. We selected ITSA because the Beijing DRG 2.0 reform had a clearly identifiable implementation month, the outcomes were available as continuous monthly hospital indicators before and after reform, and randomization or an untreated comparable hospital-level control series was not feasible within the available anonymized dataset [9,10]. By modeling the pre-reform level and trend, the immediate post-reform level change, the post-reform trend change, and monthly seasonality, ITSA is more informative than a simple pre–post comparison for separating abrupt changes from secular trends. Accordingly, using data on inpatients with CRC undergoing major surgery at a tertiary hospital in Beijing from January 2021 to October 2024, this study defined the formal implementation of DRG payment reform in April 2022 as the policy intervention point and interpreted the segmented-regression coefficients as policy-related associations.

**Figure 1:**
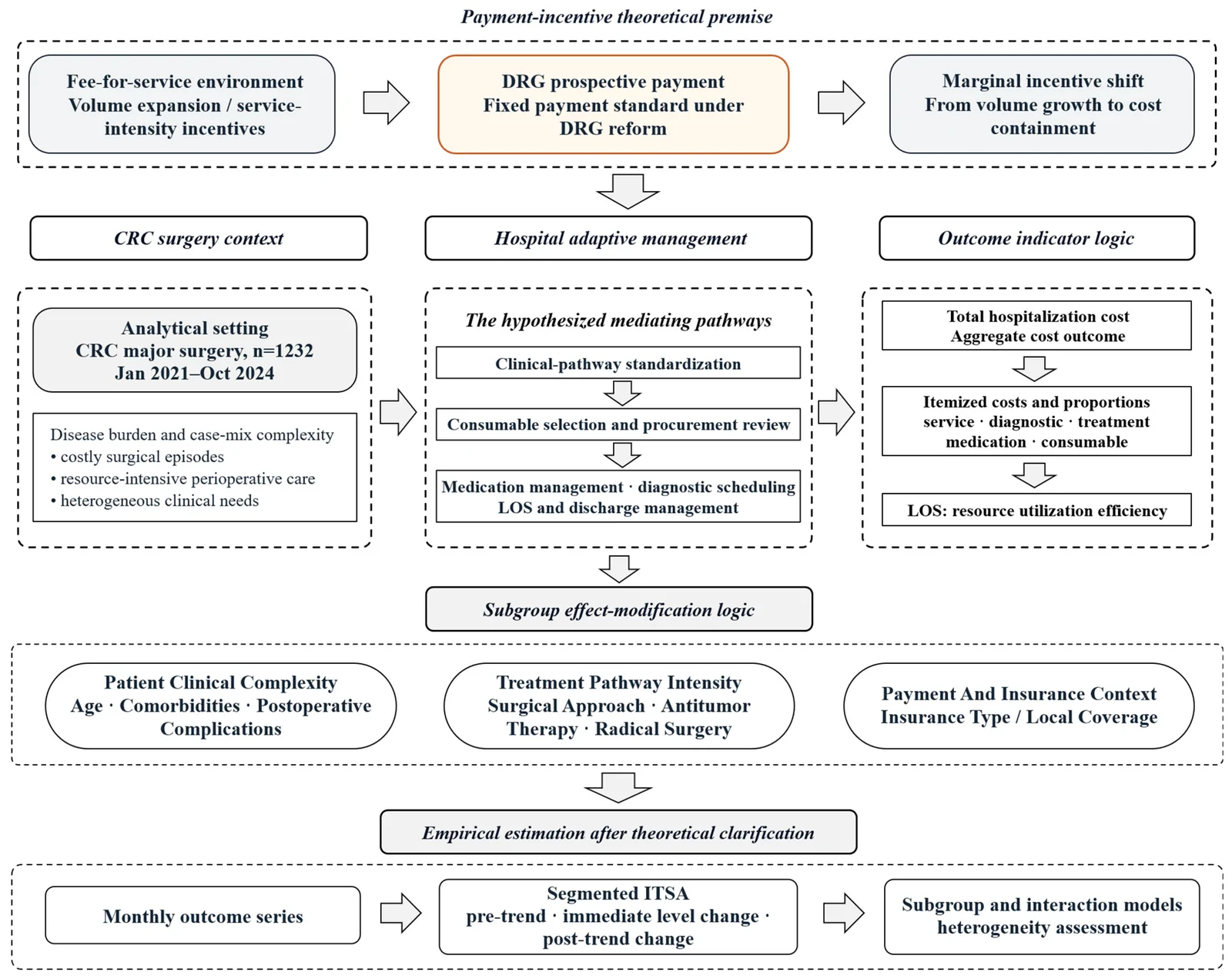
Conceptual framework linking CRC disease burden, DRG payment incentives, hospital adaptive management, outcome indicators, and subgroup effect modifiers. Different colors and box shapes distinguish conceptual domains and pathway steps.

Before empirical estimation, the theoretical linkages among the selected indicators were specified. DRG prospective payment changes hospitals’ marginal incentives from volume expansion under fee-for-service toward cost containment under a fixed payment standard, and prior economic and health system studies have described how prospective or case-based payment can affect service intensity, practice style, and inpatient resource use [11]. In CRC surgery, this incentive may operate through clinical pathway standardization, consumable selection, medication management, diagnostic scheduling, and length-of-stay management; clinical pathways have been associated with reductions in length of stay and hospital costs [12]. Total hospitalization cost reflects the final cost outcome; itemized costs and cost proportions identify the pathway of cost adjustment; and LOS captures resource utilization efficiency. Patient age, comorbidities, postoperative complications, surgical approach, recorded antitumor-treatment status, radical surgery status, and insurance type may modify these relationships through differences in clinical complexity, payment review pressure, and resource needs; comorbidity burden and surgical approach have been associated with colorectal surgery LOS, costs, and outcomes [13,14] (Figure 1).

### 2.2. Data and Sample

The study data were obtained from the Hospital Information System (HIS) of a tertiary hospital in Beijing. The hospital is a high-volume national tertiary oncology specialty hospital in China with a strong colorectal cancer surgical specialty, standardized multidisciplinary diagnosis and treatment pathways, mature HIS/DRG grouping and settlement procedures, and a patient source covering both Beijing and other provinces. In the analytical sample, local-insurance and non-local-insurance patients accounted for 26.95% and 73.05%, respectively, indicating that the cohort included patients referred from outside Beijing as well as local patients. Therefore, although the hospital cannot be regarded as a statistically representative sample of all Chinese hospitals, it provides a clinically informative and policy-relevant institutional case for evaluating DRG responses among tertiary CRC surgical patients.

The study population consisted of patients who were hospitalized at this hospital and underwent major surgery for CRC between January 2021 and October 2024. The inclusion criteria were as follows: CRC adenocarcinoma or signet-ring cell carcinoma confirmed by preoperative colonoscopic biopsy and postoperative pathology; receipt of grade III or grade IV colorectal-related major surgery, with cases classifiable into the GB39 group according to the Beijing DRG 2.0 grouping scheme and with comparable cost data; and complete medical record homepage information, clinical diagnosis and treatment information, and detailed inpatient cost records. The exclusion criteria were as follows: inconsistency between postoperative pathology and preoperative diagnosis, with final confirmation as other tumor types such as neuroendocrine tumors or lymphoma, and receipt of exploratory surgery only, stoma surgery only, or other grade II or lower-level procedures. A total of 1232 patients were ultimately included.

### 2.3. Study Variables

The primary outcome indicator was total hospitalization cost. Secondary outcomes covered sub-item medical expenses, corresponding cost proportion indicators, and hospital length of stay (LOS). Continuous variables in this study consisted of age, LOS, total hospitalization cost, as well as the absolute value and proportional share of service fees, diagnostic fees, treatment fees, western medication fees, and consumable fees. Categorical variables included demographic factors, clinical characteristics, surgical and therapeutic modalities, and insurance category. For sex, patients were divided into male and female groups; age was stratified into younger and older subgroups. Clinical indicators distinguished patients with or without comorbidities and postoperative complications. Treatment-related variables differentiated patients by recorded antitumor-treatment status before or around the surgical episode and by radical surgery status and further categorized surgical approaches as laparoscopic or open surgery. Insurance types were split into local and non-local insurance. The variables are summarized in Table 1.

### 2.4. Data Processing

First, all hospitalization cost indicators were adjusted for inflation. Given the multi-year span of the research sample, raw nominal medical expenses across different years were affected by fluctuations in general commodity prices and medical service charges, which would introduce bias into cross-period comparisons [15]. Taking 2024 as the reference base year, this study adopted Beijing’s medical care consumer price index (CPI) to deflate all nominal hospitalization expenditures into comparable real costs. All expenses from years prior to 2024 were converted using cumulative CPI adjustment coefficients between the corresponding year and the base year. The detailed CPI conversion formula and relevant parameters are presented in Table 2.

Second, raw case-level data cleaning was carried out. The initial dataset contained 1402 individual admission records. Cases missing core clinical variables or complete cost information were eliminated, leaving a final valid sample of 1232 qualified patients. Eligible individual cases were grouped by calendar month, and monthly average hospitalization expenditure and average length of stay were calculated to generate balanced monthly panel data for ITSA regression.

Third, winsorization was implemented for continuous cost variables to eliminate outlier interference. Extreme high and low values of cost indicators could severely distort monthly aggregated averages and bias model estimation results. Therefore, all continuous cost metrics were winsorized at the 1st and 99th percentiles, which effectively mitigated the impact of abnormal observations while retaining the overall distribution characteristics of the original dataset.

Lastly, missing data were handled during case-level data cleaning. The initial dataset contained 1402 admission records; 170 records (12.13%) with missing core clinical variables, incomplete cost information, or other eligibility problems were excluded during data cleaning, leaving 1232 qualified patients (87.87%). After these exclusions, the final analytical dataset contained no missing values for the variables used in the ITSA, heterogeneity analyses, or sensitivity analyses. Therefore, no linear interpolation was applied to the outcomes or covariates included in the final analyses.

### 2.5. Analysis Strategy

#### 2.5.1. Interrupted Time-Series Analysis (ITSA)

This study used a segmented regression model to estimate temporal associations between the DRG policy intervention, implemented in April 2022, and each monthly outcome indicator. The model controlled for monthly seasonality and autocorrelation using Newey–West heteroskedasticity- and autocorrelation-consistent (HAC) standard errors, a reliable correction strategy to avoid biased statistical inference from temporal serial correlation and heteroskedasticity in monthly administrative health data [16]. For the i-th indicator Y_i,t_, the model was specified as follows:

Y_i,t_ = β_0_ + β_1_ · time_t_ + β_2_ · intervention_t_ + β_3_ · time_since_t_ + ∑_m__=__1_^11^ γ_m_·month_m,t_ + ε (1)

where: 

Y_i,t_ denotes the monthly outcome indicator for the i-th indicator in month t;

t denotes the monthly time index, with January 2021 = 1, …, and October 2024 = 46;

time_t_ denotes the continuous time trend;

intervention_t_ is a policy intervention dummy variable, coded as 0 before April 2022 and 1 in April 2022 and thereafter;

time_since_t_ is the post-intervention time variable, coded as 0 before the intervention and increasing by month after the intervention;

month_m,t_ denotes 11 monthly dummy variables used to control for seasonal factors, with January as the reference month;

ε_t_ denotes the error term, allowing for autocorrelation and heteroskedasticity. Robust standard errors were calculated using the Newey–West heteroskedasticity- and autocorrelation-consistent (HAC) estimator with lag order L. The Newey–West lag order was defined as the maximum number of monthly serial-correlation lags allowed when constructing the HAC covariance matrix; thus, L = 4 allowed standard errors to be robust to autocorrelation up to four months as well as heteroskedasticity. In the final analysis, the lag order was set to L = 4.

In this model, coefficients were interpreted mainly as follows: the baseline level refers to the expected value at the time of intervention, namely April 2022, in the absence of the intervention, expressed as Y_baseline = β_0_ + β_1_·15 + seasonal adjustment (in the actual output, the predicted value for the last pre-intervention observation month was used); β_2_ represents the immediate level-change association in the first month after the intervention; and β_3_ represents the post-intervention slope-change association, namely the change in the post-intervention slope relative to the pre-intervention slope, with the new post-intervention slope equal to β_1_ + β_3_.

In addition, the counterfactual trend represents the expected trajectory of the outcome indicator under the assumption that DRG reform had not been implemented. That is, only the pre-intervention trend and seasonality were retained, without intervention terms (β_2_ = 0, β_3_ = 0). The counterfactual curve was specified as follows:

Y^counterfactual^_i,t_ = β_0_ + β_1_·time_t_ + ∑_m__=__1_^11^ γ_m_·month_m,t_ (2)

Considering that monthly time-series data may exhibit autocorrelation and heteroskedasticity, this study used Newey–West heteroskedasticity- and autocorrelation-consistent standard errors for estimation. Monthly dummy variables were also included in the model to control for seasonal fluctuations in inpatient service utilization and cost changes. The value L = 4 was selected according to residual autocorrelation diagnostics, including residual correlograms and Ljung–Box tests, which suggested that the remaining serial correlation was mainly concentrated within the first several monthly lags and was largely attenuated by lag 4. Because the Newey–West lag length is a bandwidth choice for variance estimation rather than an autoregressive model order, it was not selected using AIC/BIC. Sensitivity checks using alternative lag lengths from 1 to 6 yielded materially similar directions and significance patterns for the core DRG estimates.

All statistical hypothesis tests adopted two-tailed tests, and α = 0.05 was used as the main statistical significance threshold. Coefficients with 0.05 ≤ *p* < 0.10 were described only as marginally significant when reported with a single asterisk in exploratory analyses.

#### 2.5.2. Subgroup and Interaction Analysis

To further examine differences in the associations of DRG reform across patients and clinical subgroups, this study conducted subgroup interrupted time-series analyses. Heterogeneity analyses were carried out mainly across three dimensions: patient baseline characteristics, surgery-related characteristics, and medical insurance-related characteristics. Patient baseline characteristics included age, comorbidities, and postoperative complications. Surgery-related characteristics included surgical approach, whether radical surgery was performed, and recorded antitumor-treatment status before or around the surgical episode. Medical insurance-related characteristics included whether the patient had local medical insurance. For each subgroup, the same segmented regression model as in the main analysis was fitted using data from January 2021 to October 2024, with April 2022 as the intervention point, to estimate differences in the immediate and trend associations of DRG reform across subgroups. By comparing the direction, magnitude, and statistical significance of changes across subgroups, supplemented by formal interaction tests, we assessed whether the DRG-related temporal associations with hospitalization costs, cost structures, and length of stay differed across different types of patients.

To formally assess subgroup differences, stratified ITSA was supplemented with pooled interaction models for each binary subgroup. The pooled specification added the subgroup indicator and its interaction terms with time, intervention, and post-intervention time: Y_t_ = β_0_ + β_1_ time_t_ + β_2_ intervention_t_ + β_3_ time_since_t_ + θG + δ_1_(G × time_t_) + δ_2_(G × intervention_t_) + δ_3_(G × time_since_t_) + monthly fixed effects + ε_t_. In this framework, δ_2_ tests whether the immediate DRG level change differs between subgroups, and δ_3_ tests whether the post-reform monthly trend change differs between subgroups. These *p* values were reported to distinguish formally supported effect modification from exploratory subgroup patterns.

#### 2.5.3. Robustness Check

To examine the robustness of the temporal-association analysis, this study further used seasonal autoregressive integrated moving average (SARIMA) models to construct counterfactual forecasts as a supplementary sensitivity analysis [17,18]. The models were fitted using only pre-intervention monthly data from January 2021 to March 2022, and dynamic forecasts were generated for the post-intervention period beginning in April 2022. Forecasted values represented the expected levels of each indicator under the scenario in which DRG reform had not been implemented, and the observed post-reform values were compared with these no-reform counterfactual forecasts. Because the pre-intervention series included only 15 monthly observations and several sub-item expenditures were volatile, the SARIMA results were used as supplementary directional evidence rather than definitive validation of every outcome:

SARIMA(p, d, q)(P, D, Q)s, with s = 12 for annual seasonality in monthly data. (3)

where p, d, and q denote the non-seasonal autoregressive order, differencing order, and moving-average order, respectively; P, D, and Q denote the seasonal autoregressive order, seasonal differencing order, and seasonal moving-average order; and s represents the seasonal period. Conceptually, the model uses autoregressive and moving-average terms to capture serial dependence, differencing to address non-stationary trends, and seasonal terms to represent recurring annual monthly patterns when present. Model adequacy was assessed using stationarity checks, autocorrelation and partial autocorrelation patterns, information criteria, residual diagnostics, prediction-error metrics, and prediction-interval behavior.

Post-intervention 95% prediction-interval coverage was interpreted descriptively. It reflects the proportion of observed post-reform monthly values falling within the 95% prediction interval generated under the no-reform counterfactual scenario. Therefore, lower coverage after the intervention may indicate separation between observed post-reform values and the counterfactual no-reform trajectory. At the same time, particularly low coverage for volatile sub-item expenditures was interpreted cautiously because it may also reflect forecasting instability caused by the short pre-intervention series.

SARIMA model selection was guided by stationarity assessment, autocorrelation and partial autocorrelation patterns, information criteria, residual diagnostics, and prediction-interval behavior. Seasonal parameters were retained when supported by the available pre-intervention data and model diagnostics. Thus, the SARIMA analysis was used to triangulate whether the observed post-reform trajectories were directionally consistent with the ITSA findings, while the primary inference remained based on segmented ITSA and additional sensitivity analyses.

#### 2.5.4. COVID-19 Sensitivity Analysis

To address potential confounding from pandemic-related disruptions that overlapped with the DRG implementation period, we performed an additional sensitivity analysis. The main study period excluded 2020 to avoid the acute initial shock of the COVID-19 outbreak; however, later pandemic-control and reopening-related disturbances may still have affected hospitalization volume, admission scheduling, and case mix. Therefore, the segmented ITSA model was re-estimated after adding two COVID-19 phase indicators: a Beijing outbreak/control-period indicator for April to June 2022 and a reopening-wave/recovery indicator for January to February 2023. Monthly admission volume was also included to account for pandemic-related changes in surgical throughput. The same monthly fixed effects and Newey–West heteroskedasticity- and autocorrelation-consistent standard errors with lag order 4 were used as in the main analysis.

#### 2.5.5. Surgical-Approach-Adjusted Sensitivity Analysis

Because the proportion of laparoscopic versus open surgery differed significantly between the pre- and post-reform periods, we performed an additional sensitivity analysis to examine whether the main results were driven by changes in surgical approach. The ITSA model was re-estimated after additionally adjusting for the monthly proportion of laparoscopic surgery while retaining the original time trend, intervention indicator, post-intervention time trend, monthly fixed effects, and Newey–West HAC standard errors with lag order 4.

## 3. Results

### 3.1. Descriptive Analysis

Table 3 presents the baseline demographic and clinical characteristics of all enrolled patients. A total of 300 inpatients were included in the pre-DRG reform cohort, and 932 patients were included after DRG implementation, with an overall sample size of 1232 cases. No significant intergroup disparities in age and hospital length of stay were detected between the two periods. Total hospitalization expenses, diagnostic fees, western medication fees, and consumable fees all rose significantly after DRG reform. The proportional composition of various medical cost items differed markedly before and after the reform, whereas the proportion of western medication fees showed no statistical variation. Gender distribution, age stratification, comorbidity status, postoperative complication incidence, recorded antitumor-treatment status, and insurance composition did not differ significantly across the two groups. Statistically prominent shifts were observed in radical surgery ratio and surgical approaches. The proportion of laparoscopic operations increased substantially after DRG reform, while open surgery and non-radical surgery rates declined correspondingly.

We additionally computed an exploratory Spearman rank correlation analysis among the monthly core indicators (Table S1). The correlation matrix showed that total hospitalization cost was strongly correlated with consumable fees (rho = 0.907, *p* < 0.001) and western medication fees (rho = 0.771, *p* < 0.001), supporting the decomposition of total expenditure into major cost components. Length of stay was positively correlated with western medication fees (rho = 0.658, *p* < 0.001) and service fees (rho = 0.510, *p* < 0.001), suggesting that resource use intensity and inpatient duration were related.

### 3.2. Main Analysis: Temporal Associations with Hospitalization Costs and Length of Stay

**Figure 2:**
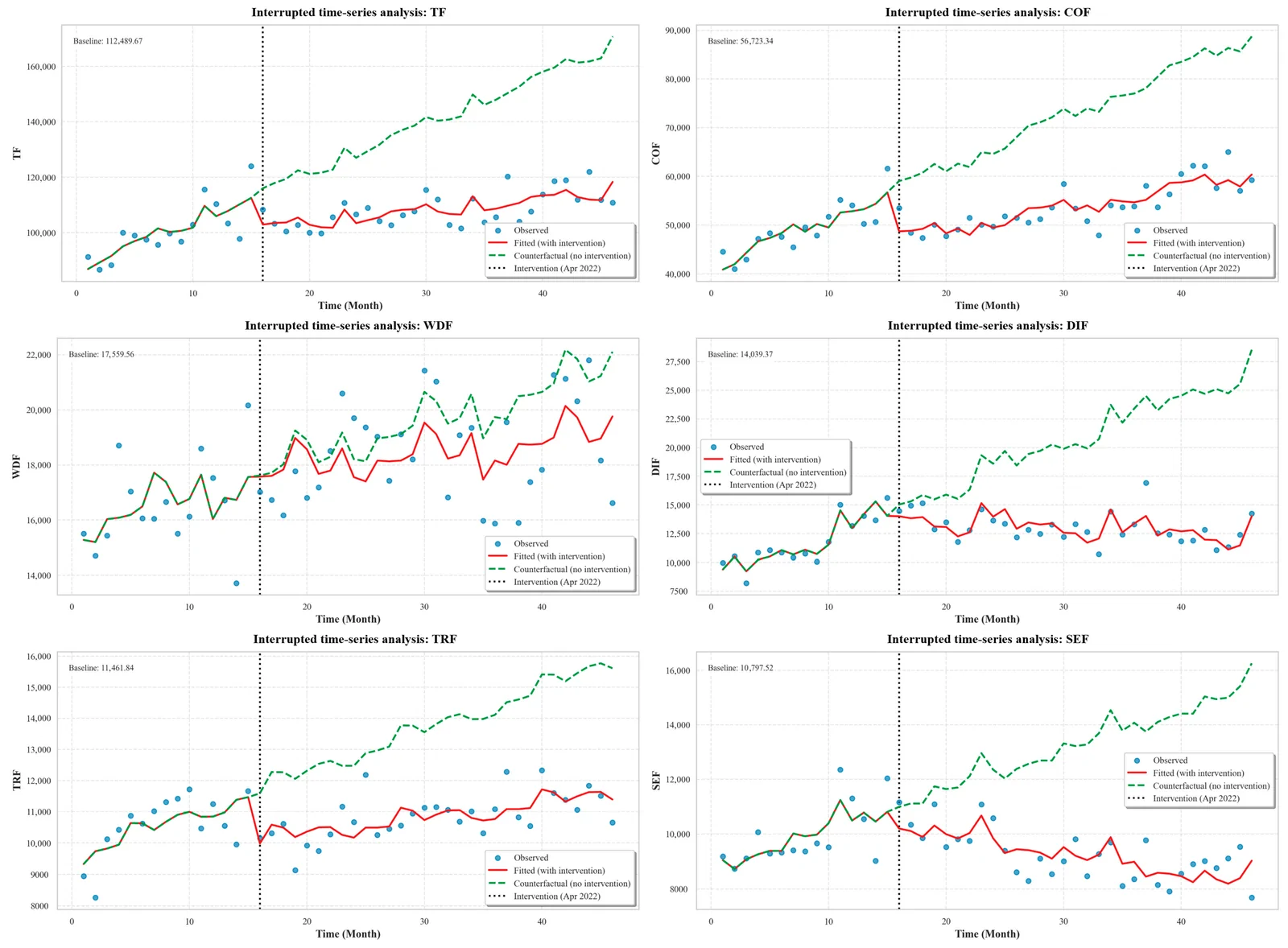
Interrupted time-series analysis of total medical expense and five sub-category inpatient costs before and after the DRG payment reform.

All coefficient values, confidence intervals, and significance levels for total and sub-item fees are listed in Table 4, while monthly fluctuation trajectories of each absolute cost indicator are visualized in Figure 2. Results revealed that the implementation of Beijing’s DRG 2.0 prospective payment reform was temporally associated with statistically significant, multi-faceted changes in absolute hospitalization costs, internal cost composition, and inpatient service efficiency among surgical CRC patients.

Firstly, two distinct cost-reduction associations with total expenditure were observed in the segmented regression model after policy implementation. An immediate decrease in total hospitalization costs by 13,111.73 CNY emerged at the intervention month (β_2_ = −13,111.73, *p* < 0.001), indicating an abrupt level change temporally associated with the formal launch of DRG bundled payment. Beyond the intercept shift, a persistent downward trajectory formed in the post-reform period: monthly total hospitalization costs declined by an additional 1312.60 CNY each month (β_3_ = −1312.60, *p* < 0.001), which indicates that the cost-control trend became stronger after reform relative to the counterfactual pre-reform slope.

Further decomposition of sub-item absolute fees identifies medical consumable fees (COF) as the core driver of overall total-cost decline. COF exhibits both a statistically significant immediate level drop and a sustained monthly downward trend, with both coefficients significant at the 0.001 level, marking the largest magnitude of expenditure cut across all fee categories. Heterogeneous trends are observed for remaining cost items. western medication fees (WDF) show no statistically meaningful shifts in either immediate level or long-term monthly trend after reform. Diagnostic fees (DIF) and nursing service fees (SEF) display non-significant instantaneous level changes upon policy implementation yet record statistically significant monthly declines throughout the post-intervention stage. Treatment fees (TRF) showed an immediate reduction with marginal statistical significance (*p* < 0.05), while its long-term monthly declining trend lacks statistical significance.

**Figure 3:**
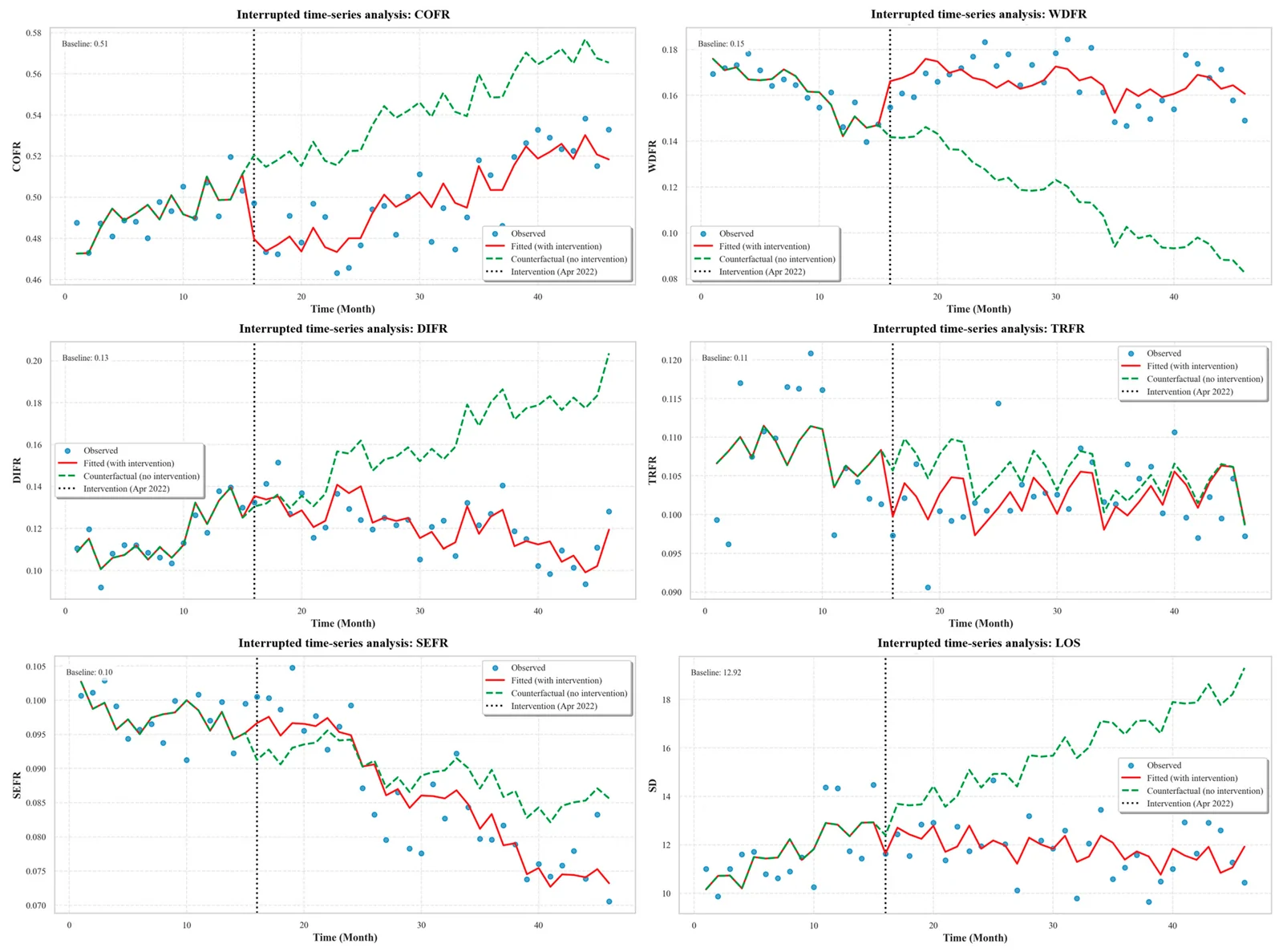
Interrupted time-series analysis of five sub-item medical cost ratios and inpatient length of stay before and after the DRG payment reform.

Second, the dynamic evolution of each cost proportion over the study period is displayed in Figure 3. Corresponding to the sharp absolute decline in consumable expenditures, the proportion of consumable fees within total hospitalization costs (COFR) registers an immediate 4.1-percentage-point drop (*p* < 0.001), whereas its monthly trend change remains negligible and non-significant. A prominent structural contrast emerges for western medication fees. Despite stable absolute WDF volume, the proportional share of western medications (WDFR) rises instantaneously by 2.4 percentage points following reform (*p* < 0.001). For diagnostic fees (DIFR) and service fees (SEFR), their proportional shares sustain continuous significant monthly declines after reform, consistent with the falling absolute values of DIF and SEF. The proportion of treatment fees (TRFR) shows no significant instantaneous or long-term trend variations, with its relative weight in total expenditures remaining largely unchanged before and after policy implementation.

To further examine the rigidity of western medication fees, the index hospitalization expenditures analyzed in this study were surgery-related hospitalization costs and did not directly include targeted therapy, immunotherapy, or other systemic antitumor drug costs as major components of the surgical admission. Therefore, we did not attribute WDF rigidity to direct in-hospital use of high-cost antitumor agents. Instead, we used the available recorded antitumor-treatment status only as an exploratory indicator of treatment background that might indirectly influence surgical hospitalization costs through patient physical condition, operative difficulty, postoperative complications, or LOS. The proportion of patients with recorded antitumor therapy increased from 25.33% before DRG reform to 31.01% after reform, although this difference did not reach conventional statistical significance (χ^2^ *p* = 0.072). After additionally adjusting the ITSA model for the monthly proportion of patients with recorded antitumor therapy, the absolute WDF still showed no significant immediate or trend decrease (β_2_ = −9.32 CNY; 95% CI, −2584.02 to 2565.37; *p* = 0.994; β_3_ = −86.34 CNY per month; 95% CI, −244.95 to 72.27; *p* = 0.275), whereas the proportional share of western medication fees remained significantly increased (β_2_ = 0.0246; 95% CI, 0.0110 to 0.0383; *p* < 0.001; β_3_ = 0.0017 per month; 95% CI, 0.0007 to 0.0027; *p* = 0.002). These findings suggest that the rising WDFR was more likely related to the denominator effect caused by reductions in other cost items and to the clinical rigidity of perioperative medication needs than to direct targeted therapy or immunotherapy costs during the surgical hospitalization.

Third, evaluation focuses on inpatient service efficiency measured by length of stay (LOS). The immediate level change coefficient for LOS equals −0.761 days (*p* > 0.05), signifying no abrupt reduction in hospitalization days within the first month of DRG execution. Nevertheless, a robust statistically significant negative trend emerges post-reform: average length of stay shortens by 0.22 days per month (β_3_ = −0.221, *p* < 0.001). Full regression statistics for length of stay are provided in Table 4, and the temporal trend of average hospitalization days is illustrated in Figure 3.

### 3.3. Heterogeneity Analysis

Subgroup segmented regression outputs, supplemented by formal interaction tests, reveal disparities in the immediate and trend associations of DRG reform with total hospitalization expenditure (TF) and length of stay (LOS), as shown in Table 5 and Table 6. We therefore interpreted heterogeneity based on both subgroup-specific estimates and the corresponding interaction-test *p* values.

The interaction results are summarized in Table 6. Statistically supported association modification was observed for LOS trend differences by age, total-cost trend and LOS patterns by postoperative complication status, total-cost patterns by surgical approach, total-cost trend differences by radical surgery status, and LOS trend differences by recorded antitumor-treatment status. In contrast, subgroup patterns for binary comorbidity status and medical insurance type were interpreted cautiously because their interaction tests were not statistically significant.

#### 3.3.1. Heterogeneity by Patient Clinical Characteristics

Distinct clinical conditions created differentiated space for hospitals to adjust medical resource allocation under DRG constraints, but formal interaction tests indicated that the strength of evidence varied across dimensions. Age stratification generated a statistically significant interaction for the LOS trend association, with younger patients showing a stronger sustained decline in LOS than older patients; however, the between-age interaction for total-cost associations was not statistically significant. Binary comorbidity status showed different point estimates but did not demonstrate statistically significant interaction patterns. By contrast, postoperative complications showed clear association modification: patients with postoperative complications experienced a larger immediate LOS reduction and a stronger monthly total-cost decline than those without postoperative complications.

#### 3.3.2. Surgical and Treatment Modalities

Operative approaches and treatment modalities produced divergent trajectories for total costs and service efficiency. Formal interaction tests supported stronger immediate and trend total-cost associations for open surgery than for laparoscopic surgery, while the LOS interaction by surgical approach approached but did not reach conventional statistical significance. Radical surgery status showed a significant interaction for the total-cost trend association, indicating stronger sustained cost compression among non-surgical curative cases than among patients receiving surgical curative treatment. Recorded antitumor-treatment status showed a significant interaction for the LOS trend association, suggesting that treatment background was associated with a different post-reform LOS trajectory, although total-cost interactions for antitumor-treatment status were not statistically significant.

#### 3.3.3. Insurance Attributes and Systemic Factors

Insurance-related settlement factors may influence hospital billing and management behavior; however, the formal interaction tests did not provide strong statistical evidence that DRG-related associations differed by local versus non-local insurance status. Therefore, the observed stronger sustained cost-reduction trend among patients with non-local insurance was interpreted as an exploratory pattern rather than definitive association modification.

### 3.4. Robustness Checks

Counterfactual SARIMA forecasting was used as a supplementary sensitivity analysis. We used pre-reform monthly data from January 2021 to March 2022 to generate no-reform counterfactual forecasts for the post-intervention period. Comparative statistics between post-reform observed monthly averages and counterfactual predicted values are presented in Table 7.

The SARIMA counterfactual analysis showed that observed post-reform values for the main outcomes were generally lower than the no-reform counterfactual forecasts, but these results were interpreted as supplementary and directional rather than definitive validation of every sub-item. For total hospitalization cost, the post-reform mean observed value was 108,331.54 CNY compared with a counterfactual predicted value of 167,949.73 CNY, yielding an average difference of −59,618.19 CNY. For consumable fees, the corresponding observed and predicted values were 53,883.81 CNY and 84,490.74 CNY, respectively, with an average difference of −30,606.94 CNY. LOS was also lower than the counterfactual prediction (11.84 vs. 18.15 days). We note that low post-intervention 95% prediction-interval coverage should not be read simply as poor in-sample forecasting accuracy because the forecasts represent a no-reform counterfactual scenario.

### 3.5. Sensitivity Analysis Accounting for COVID-19 Disruptions

The COVID-/volume-adjusted sensitivity analysis showed that the core findings were not materially changed (Table S2 and Figure S1). After additional adjustment for the Beijing pandemic-control period, the reopening-wave/recovery period, and monthly admission volume, total hospitalization costs remained significantly lower immediately after DRG implementation (β_2_ = −14,872.51 CNY; 95% CI, −21,258.34 to −8486.68; *p* < 0.001), with a sustained monthly downward trend (β_3_ = −1238.98 CNY; 95% CI, −1850.30 to −627.67; *p* < 0.001). Consumable fees also remained the major cost component associated with reduction (β_2_ = −10,867.36 CNY; 95% CI, −14,090.70 to −7644.01; *p* < 0.001; β_3_ = −519.81 CNY per month; 95% CI, −838.54 to −201.08; *p* = 0.002). For LOS, the immediate level change remained non-significant, whereas the post-reform monthly trend continued to decline significantly (β_3_ = −0.24 days per month; 95% CI, −0.38 to −0.10; *p* = 0.002). These results support the stability of the main ITSA conclusions after accounting for major COVID-19-related disturbances.

### 3.6. Sensitivity Analysis Adjusting for Surgical Approach

The surgical-approach-adjusted sensitivity analysis also supported the stability of the main conclusions (Table S3). After adjustment for the monthly proportion of laparoscopic surgeries, total hospitalization costs remained significantly lower immediately after DRG implementation (β_2_ = −13,602.50 CNY; 95% CI, −20,628.00 to −6576.99; *p* < 0.001) and maintained a significant downward monthly trend (β_3_ = −1184.13 CNY per month; 95% CI, −1787.81 to −580.46; *p* < 0.001). Consumable fees remained significantly reduced both immediately and over time (β_2_ = −10,868.20 CNY; 95% CI, −13,750.33 to −7986.06; *p* < 0.001; β_3_ = −457.52 CNY per month; 95% CI, −773.76 to −141.27; *p* = 0.006). The immediate change in LOS remained non-significant, whereas the post-reform monthly trend in LOS remained significantly downward (β_3_ = −0.20 days per month; 95% CI, −0.31 to −0.08; *p* = 0.002). These findings suggest that the main results were not driven solely by the shift from open to laparoscopic surgery.

## 4. Discussion

### 4.1. Principal Findings

The main findings can be summarized in four aspects: (1) DRG reform was temporally associated with lower total hospitalization costs and a steeper downward cost trend; (2) the decrease was mainly linked to consumable-fee compression rather than uniform reduction across all cost categories; (3) LOS showed a sustained downward trend, although post-discharge quality could not be assessed; and (4) selected clinical and treatment subgroups showed different association patterns. All interpretations are framed as temporal associations because the study used a single-center design without a parallel control group.

First, the DRG prospective payment framework was associated with lower total hospitalization expenditure at both the immediate level and the post-reform monthly trend. This finding is broadly consistent with prior evidence that case-based payment reforms are often accompanied by restrained inpatient expenditure, including evidence from Beijing DRG and other payment reform studies [6,19,20]. However, our estimate should not be interpreted as an isolated causal effect of DRG reform because concurrent policies and case-mix changes may also have contributed to the observed trajectory.

Second, the cost decrease was mainly linked to the compression of consumable fees. This focused cost-control pathway is clinically plausible in CRC surgery because staplers, anastomosis materials, laparoscopic devices, and other surgical consumables represent a large and relatively manageable share of surgery-related inpatient expenditure. In contrast, western medication fees during the index surgical hospitalization may be less compressible because they mainly reflect perioperative anti-infective therapy, anesthesia-related medications, nutritional support, albumin, analgesics, and medications for comorbid conditions that are tied to clinical need, rather than direct use of targeted therapy or immunotherapy in the surgical admission. Therefore, the observed cost-structure shift likely reflects selective hospital management responses under DRG constraints rather than homogeneous reductions in all services [21,22]. Earlier colorectal-cancer surgical studies also linked perioperative resource factors and DRG use with hospital charges and LOS [23,24].

Third, the internal cost composition changed after reform. The proportion of consumable fees declined, whereas the proportion of western medication fees increased despite no significant absolute WDF decrease. This pattern may partly reflect a denominator effect caused by larger reductions in consumable and other adjustable fees, while perioperative medication use remained clinically rigid. The exploratory antitumor-treatment-status-adjusted analysis was used only to examine whether treatment background might indirectly affect surgery-related hospitalization costs through patient condition, operative difficulty, postoperative complications, or LOS. Because the index hospitalization costs analyzed here were surgery-related and drug-level preoperative regimen data were unavailable, targeted therapy or immunotherapy should not be interpreted as a directly verified or direct-cost explanation.

Mechanistically, prospective payment may encourage hospitals to strengthen clinical pathway management, preoperative planning, internal cost accounting, consumable selection, procurement review, and discharge planning. These mechanisms are particularly relevant for CRC surgery because high-value consumables and LOS-related resource use are more amenable to management than clinically necessary medications. The sustained LOS decline is consistent with improved bed-turnover efficiency. Nevertheless, because readmissions to outside hospitals, especially among non-local patients, may not be captured completely or promptly, unchanged post-discharge quality cannot be definitively demonstrated.

Concurrent policy effects remain important. During the study period, DRG implementation overlapped with COVID-19-related service disruptions, centralized procurement and governance of high-value medical consumables, medical service price and hospital cost-accounting reforms, and hospital-level clinical pathway and quality-management initiatives. Although COVID-/volume-adjusted and surgical-approach-adjusted sensitivity analyses supported the direction of the main findings, residual time-varying confounding cannot be fully excluded.

### 4.2. Multi-Dimensional Heterogeneity in Policy-Related Associations

Beyond the overall average associations of DRG payment reform, subgroup ITSA estimations and formal interaction tests showed that the magnitude of cost containment and efficiency improvement varied across selected patient clinical profiles and treatment modalities. The discussion focuses on subgroup patterns supported by formal interaction tests and interprets unsupported patterns as exploratory.

First, age was an important source of heterogeneity for LOS rather than for total-cost effects. Younger patients showed a significantly stronger sustained decline in length of stay, whereas older patients exhibited more limited room for efficiency improvement. This finding is consistent with prior age-stratified evidence from DIP reform. Chen et al. found that after DIP implementation, length of stay decreased significantly among younger and young-old inpatients, while total costs increased among older and oldest-old patients [25]. Atypical symptoms may delay diagnosis for younger CRC patients, whereas limited physiological reserve and heavy comorbidities likely prolong elderly patients’ recovery [26]. Prior research suggested DRG weights may overemphasize age relative to surgical complexity [27]. Relevant public health interrupted time-series studies also indicated stratified policy effects by age and socioeconomic status [28,29]. Our results further demonstrate that age-related efficiency responses may also exist within a single oncology surgical population.

Second, postoperative complications, rather than binary comorbidity status, showed the clearest formally supported clinical association modification. Patients with postoperative complications experienced a larger immediate reduction in length of stay and a stronger downward trend in total costs, indicating that high-resource-consuming cases may have greater potential for process optimization under DRG-based pathway management. Nevertheless, this should not be interpreted as indicating unlimited compressibility of complex cases. For patients with multiple comorbidities or postoperative complications, a considerable share of resource use is clinically necessary. Comorbidities and postoperative complications could push patients’ LOS and costs above DRG expectations and bring potential financial pressure to hospitals [30]. Excessive cost-control pressure under a uniform payment standard may therefore increase the risk of under-provision, premature discharge, or avoidance of high-risk patients.

Third, divergent policy-related association patterns existed across surgical and treatment-related modalities. Open radical resection showed stronger total-expenditure reductions after DRG implementation than laparoscopic surgery, whereas minimally invasive laparoscopic surgery showed more gradual long-term optimization of inpatient duration. Radical surgery status modified the total-cost trend association, and recorded antitumor-treatment status modified the LOS trend association, suggesting that treatment background and clinical condition may influence the space available for hospitals to adjust costs and bed turnover. However, this should not be interpreted as direct evidence that targeted or immunotherapeutic drugs changed index surgical hospitalization costs. If preoperative antitumor medication affected surgery-related hospitalization costs, its influence would likely have occurred indirectly through patient physiological condition, surgical difficulty, postoperative complications, and LOS. Similar oncology-focused evidence has also indicated that DRG reform can alter cost homogeneity among malignant-tumor treatments [31]. Comparative cost analyses have consistently shown that laparoscopic colorectal resection, despite higher intraoperative consumable costs, generates substantial overall savings through reduced LOS and lower complication-related costs compared with open surgery [32,33].

Fourth, medical insurance attributes were retained as an exploratory policy-relevant dimension, but the formal interaction tests did not confirm statistically significant association modification by local versus non-local insurance status. The stronger point-estimated cost-reduction trend among patients with non-local insurance may reflect stricter cross-regional settlement review and stronger pressure for standardized billing, but this explanation should be interpreted cautiously. Prior research on DIP reform also found that payment reform associations varied across hospital ownership and hospital level, with inconsistent patterns in total expenditure, length of stay, and mortality across different institutions [34,35]. Compared with those institution-level studies, our study suggests that future analyses should examine clinical and insurance-related heterogeneity within single disease populations using prospectively defined payment-risk indicators, DRG weights, disease severity scores, tumor stage, or pre-admission clinical risk measures.

### 4.3. Policy Implications

The findings of this study have several implications for the refinement of DRG payment reform in complex oncology care. First, stratified DRG payment standards and exceptional case settlement mechanisms should be strengthened for CRC patients with high clinical complexity. Medical insurance regulators should consider incorporating age, comorbidity burden, complication status, surgical complexity, and treatment background into payment adjustment mechanisms. Second, clinical pathway management should be differentiated according to surgical approach and treatment modality. For open surgery, cost-control efforts may focus on standardizing perioperative consumable use and reducing unnecessary high-value material consumption. For laparoscopic surgery, management should emphasize accelerated recovery, postoperative monitoring, discharge planning, and bed-turnover efficiency. Third, regulators should monitor cost-structure changes dynamically to distinguish reasonable cost optimization from inappropriate cost shifting. Routine monitoring should not focus only on total costs and length of stay but should also track changes in drugs, consumables, diagnostics, treatment fees, service fees, postoperative complications, and unplanned readmissions within the same DRG whenever cross-institution data are available. The surgical-approach-adjusted sensitivity analysis further indicated that the observed reductions in total costs, consumable fees, and LOS trends were not explained solely by the changing proportion of laparoscopic surgery.

### 4.4. Limitations

This study has several limitations. First, as a single-center study based on a high-volume national tertiary oncology specialty hospital in Beijing, the sample has policy relevance for tertiary CRC surgical care. The hospital has standardized multidisciplinary diagnosis and treatment pathways, a strong CRC surgical specialty, and admits a substantial number of patients from outside Beijing, as reflected by the high proportion of patients with non-local insurance in the analytical sample. Nevertheless, it remains a single institutional case rather than a statistically representative sample of all Beijing or Chinese hospitals; therefore, the generalizability of the findings to other regions, hospital tiers, general hospitals, and different payment environments should be interpreted cautiously. Second, the lack of a parallel control group is an important methodological limitation. A single-group ITSA can adjust for baseline level, secular trend, seasonality, and autocorrelation, but it cannot fully separate DRG-related temporal changes from other time-varying factors such as COVID-19 disruptions, centralized procurement, clinical pathway reforms, coding practice changes, admission criteria, or unobserved case-mix shifts. For this reason, the results should be interpreted as within-hospital temporal associations rather than definitive causal effects. Third, constrained by data availability, this study could not fully control for tumor stage, pathological risk classification, molecular subtype, detailed preoperative antitumor regimen, exact DRG weights, or standardized disease severity scores, which may introduce residual confounding. Future studies should use multi-center data, controlled interrupted time-series or difference-in-differences designs, and full-cycle cost-quality indicators to more rigorously evaluate the net associations of DRG reform among complex oncology patients. Although ASA classification and the Charlson Comorbidity Index would have allowed more nuanced severity adjustment, these measures could not be constructed from the available anonymized variables.

## 5. Conclusions

The DRG payment reform was associated with significant containment of surgery-related hospitalization costs and a sustained reduction in the monthly trend of LOS among CRC patients, and it also slowed the overall growth rate of inpatient medical expenditures. The findings suggest that the reduction in CRC inpatient expenditure was more closely related to the compression of consumable fees, while the rising WDFR may indicate hospital adaptive cost-structure adjustments under fixed DRG payment constraints and the clinical rigidity of perioperative medication needs. Moreover, our results further highlight that DRG-related temporal associations may vary across both clinical and systemic subgroups within a single disease population. Therefore, a uniform DRG standard is insufficient for complex oncology patients, and future reform should incorporate stratified payment mechanisms, robust exceptional case policies, and quality monitoring using full-cycle readmission and follow-up data to balance cost containment with high-quality and accessible care.

## Figures and Tables

**Table 1:** Variable definition.

Variable Name	Variable Grouping/Definition	Code
Sex	Patient gender, divided into two groups: male; female	/
Age	Age stratification standard: ≤65 years old (younger age); >65 years old (older age)	/
Comorbidities	Two groups: with comorbidities/without comorbidities; comorbidities include hypertension, diabetes mellitus, heart disease, cerebrovascular disease, hepatitis, and other chronic systemic illnesses	/
Postoperative complications	Two categories: with post-op complications/non-post-op complications	
Surgical approach	Two groups: laparoscopic surgery; open surgery	/
Antitumor therapy	Two categories: with recorded antitumor-treatment status before/around the surgical episode/without recorded antitumor-treatment status; detailed drug regimens and drug classes were unavailable, and targeted/immunotherapy costs were not treated as direct index-surgical-admission cost drivers	
Radical surgery status	Two categories: with surgical curative resection; non-surgical curative treatment	
Medical insurance type	Merged grouping after standardization: local insurance; non-local insurance	
Total hospitalization expenditure	Total hospitalization expenditure of the patient	TF
Diagnostic fees	Including ultrasound, pathology, radiology, laboratory testing fees, etc.	DIF
Proportion of diagnostic fees	Diagnostic fees as a proportion of total costs	DIFR
Consumable fees	Including disposable medical material fees for examinations, treatments, surgery, and interventional procedures	COF
Proportion of consumable fees	Consumable fees as a proportion of total costs	COFR
Western medication fees	Including western medication fees, antimicrobial drug fees, albumin product fees, Chinese patent medicine fees, etc.	WDF
Proportion of western medication fees	Western medication fees as a proportion of total costs	WDFR
Medical treatment fees	Including consultation, surgery, anesthesia, general examination and treatment, interventional treatment, and rehabilitation treatment fees	TRF
Proportion of medical treatment fees	Medical treatment fees as a proportion of total costs	TRFR
Service fees	Costs incurred by patients for receiving nursing services	SEF
Proportion of service fees	Service fees as a proportion of total costs	SEFR
Length of stay	Total inpatient days of index hospitalization, unit: days	LOS

**Table 2:** CPI-based cost adjustment.

Year	CPI	Original Cost	Adjusted Cost
2024	99.80%	C1	C1′ = C1
2023	100.20%	C2	C2′ = C2 × 99.80%
2022	100.70%	C3	C3′ = C3 × 99.80% × 100.20%
2021	99.80%	C4	C4′ = C4 × 99.80% × 100.20% × 100.70%

**Table 3:** Sample characteristics of patients undergoing CRC surgery before and after DRG payment reform (*n* = 1232).

Variable Name	All	Before DRG Reform	After DRG Reform	*p* Value
*Discharge cases*	1232	300	932	
*Continuous variable, mean (SD)*				
age	60.20 (±11.00)	59.55 (±10.95)	60.41 (±11.02)	0.24
Total cost	106,873.51 (±27,494.26)	99,412.06 (±26,905.29)	109,275.26 (±27,263.92)	<0.001
Service fees	9441.03 (±4664.49)	9896.60 (±4919.02)	9294.39 (±4572.64)	0.05
Proportion of service fees	0.09 (±0.02)	0.10 (±0.02)	0.08 (±0.02)	<0.001
Diagnostic fees	12,639.13 (±5378.28)	11,387.54 (±5280.29)	13,042.00 (±5350.25)	<0.001
Proportion of diagnostic fees	0.12 (±0.04)	0.11 (±0.04)	0.12 (±0.04)	0.01
Medical treatment fees	10,832.43 (±2619.92)	10,540.10 (±2513.73)	10,926.53 (±2647.65)	0.03
Proportion of medical treatment fees	0.10 (±0.02)	0.11 (±0.03)	0.10 (±0.02)	<0.001
Western medication fees	18,164.73 (±9500.83)	16,627.36 (±8691.28)	18,659.60 (±9699.79)	<0.001
Proportion of western medication fees	0.17 (±0.04)	0.16 (±0.04)	0.17 (±0.04)	0.66
Consumable fees	52,990.41 (±12,928.52)	48,361.93 (±11,417.10)	54,480.26 (±13,039.11)	<0.001
Proportion of consumable fees	0.50 (±0.06)	0.49 (±0.06)	0.50 (±0.06)	0.01
Length of stay	11.84 (±6.74)	11.69 (±6.67)	11.89 (±6.77)	0.65
*Categorical variable*				
Sex				
Male	765.0 (62.09)	198.0 (66.0)	567.0 (60.84)	0.12
Female	467.0 (37.91)	102.0 (34.0)	365.0 (39.16)	0.12
Age group				
Younger age	740.0 (60.06)	183.0 (61.0)	557.0 (59.76)	0.75
Older age	492.0 (39.94)	117.0 (39.0)	375.0 (40.24)	0.75
Patient clinical characteristics				
With comorbidities	692.0 (56.17)	162.0 (54.0)	530.0 (56.87)	0.42
Without comorbidities	540.0 (43.83)	138.0 (46.0)	402.0 (43.13)	0.42
With post-op complications	136.0 (11.04)	42.0 (14.0)	94.0 (10.09)	0.07
Non-post-op complications	1096.0 (88.96)	258.0 (86.0)	838.0 (89.91)	0.07
Surgical and treatment modalities				
With antitumor therapy	365.0 (29.63)	76.0 (25.33)	289.0 (31.01)	0.07
Non-antitumor therapy	867.0 (70.37)	224.0 (74.67)	643.0 (68.99)	0.07
With surgical curative	1074.0 (87.18)	280.0 (93.33)	794.0 (85.19)	<0.001
Non-surgical curative	158.0 (12.82)	20.0 (6.67)	138.0 (14.81)	<0.001
Laparoscopic surgery	1109 (90.02)	241 (80.33)	868 (93.13)	<0.001
Open surgery	123 (9.98)	59 (19.67)	64 (6.87)	<0.001
Insurance type				
Local insurance	332 (26.95)	91 (30.33)	241 (25.86)	0.15
Non-local insurance	900 (73.05)	209 (69.67)	691 (74.14)	0.15

Note: Italicized entries indicate table section headings.

**Table 4:** ITSA results for temporal associations with total fee and itemized fee outcomes.

Outcome	Baseline	Instantaneous Effect (β_2_)	Trend Change (β_3_)	95% CI (IE)	95% CI (TC)
TF	112,489.67	−13,111.73 *** (SE = 3250.55)	−1312.60 *** (SE = 268.62)	[−19,741.26, −6482.20]	[−1860.44, −764.75]
COF	56,723.34	−10,322.94 *** (SE = 1466.71)	−600.24 *** (SE = 145.94)	[−13,314.32, −7331.57]	[−897.89, −302.59]
COFR	0.51	−0.04 *** (SE = 0.01)	−0.00 (SE = 0.00)	[−0.06, −0.03]	[−0.00, 0.00]
WDF	17,559.56	−37.73 (SE = 1237.25)	−77.04 (SE = 69.53)	[−2561.12, 2485.66]	[−218.85, 64.76]
WDFR	0.15	0.02 *** (SE = 0.01)	0.00 *** (SE = 0.00)	[0.01, 0.04]	[0.00, 0.00]
DIF	14,039.37	−1023.12 (SE = 520.12)	−449.92 *** (SE = 46.12)	[−2083.92, 37.68]	[−543.98, −355.86]
DIFR	0.13	0.01 (SE = 0.01)	−0.00 *** (SE = 0.00)	[−0.01, 0.02]	[−0.00, −0.00]
TRF	11,461.84	−1601.64 * (SE = 679.43)	−87.45 (SE = 60.56)	[−2987.34, −215.93]	[−210.96, 36.06]
TRFR	0.11	−0.01 (SE = 0.01)	0.00 (SE = 0.00)	[−0.02, 0.01]	[−0.00, 0.00]
SEF	10,797.52	−788.246 * (SE = 353.606)	−214.78 *** (SE = 37.37)	[−1509.43, −67.06]	[−291.00, −138.57]
SEFR	0.10	0.01 * (SE = 0.00)	−0.00 * (SE = 0.00)	[0.00, 0.01]	[−0.00, −0.00]
LOS	12.92	−0.76 (SE = 0.59)	−0.22 *** (SE = 0.05)	[−1.95, 0.43]	[−0.32, −0.12]

Note: 1. *** *p* < 0.001 and * *p* < 0.05. 2. The regressions include constant terms but are not reported.

**Table 5:** Heterogeneous temporal associations of DRG payment reform stratified by clinical, surgical, and medical insurance factors.

Dimension and Subgroup	TF Immediate Effect	TF Trend Effect	LOS Immediate Effect	LOS Trend Effect
**Age Group**				
Younger Age	−13,658.00 ***	−1673.85 ***	−1.25 *	−0.34 ***
Older Age	−14,455.81 ***	−1046.78 ***	−0.3	−0.09 **
**Comorbidity Status**				
Without Comorbidities	−8534.87	−1355.57 ***	−0.51	−0.24 **
With Comorbidities	−16,729.52 **	−1307.19 **	−1.01	−0.20 **
**Postoperative Complications**				
With Post-op Complications	−45,507.3	−5149.37 **	−6.95 *	−0.82 **
Non-Post-op Complications	−5689.15 **	−545.52 ***	0.37	−0.11 ***
**Surgical Approach**				
Open Surgery	−18,959.37 **	−1513.28 **	0.82	−0.34 **
Laparoscopic Surgery	−9770.08 ***	−884.69 ***	−0.2	−0.14 ***
**Radical Surgery Status**				
With Surgical Curative	−13,159.02 ***	−1253.63 ***	−1.07 *	−0.24 ***
Non-surgical Curative	−25,100.09 *	−5661.37 **	−0.12	−0.56
**Antitumor Therapy**				
With Antitumor Therapy	−8985.98	−952.13	−2.23	−0.50 ***
Non-Antitumor Therapy	−15,703.25 ***	−1423.89 ***	−0.09	−0.13 **
**Medical Insurance Type**				
Local Insurance	−11,522.7	−749.2	−2.57	−0.22
Non-local Insurance	−15,515.4	−1720.54	−0.6	−0.22

Note: 1. *** *p* < 0.001, ** *p* < 0.01, and * *p* < 0.05. 2. The regressions include constant terms but are not reported. 3. Bold text indicates subgroup-dimension headings.

**Table 6:** Formal interaction tests for subgroup differences in DRG-related temporal associations.

Contrast	Outcome	*p* for IE Interaction	*p* for Trend Interaction
Older Age vs. Younger Age	TF	0.852	0.282
	LOS	0.332	**0.001**
With Comorbidities vs. Without Comorbidities	TF	0.209	0.919
	LOS	0.634	0.662
With Post-op Complications vs. Non-Post-op Complications	TF	0.133	**0.009**
	LOS	**0.016**	**0.006**
Open Surgery vs. Laparoscopic Surgery	TF	**0.014**	**0.046**
	LOS	0.148	**0.059**
Non-surgical Curative vs. With Surgical Curative	TF	0.264	**0.007**
	LOS	0.534	0.726
With Antitumor Therapy vs. Non-Antitumor Therapy	TF	0.480	0.313
	LOS	0.250	**0.010**
Non-local Insurance vs. Local Insurance	TF	0.695	0.252
	LOS	0.242	0.907

Note: IE denotes the immediate level-change coefficient after DRG implementation; trend denotes the post-intervention monthly trend-change coefficient. *p* values are from pooled interrupted time-series models with subgroup × intervention and subgroup × post-intervention-time interaction terms.

**Table 7:** SARIMA counterfactual sensitivity analysis.

Outcome	Mean Actual	Mean Predicted	Avg Effect	95% PI Coverage (%)
TF	108,331.54	167,949.73	−59,618.19	93.55
SEF	9261.99	15,380.30	−6118.30	100.00
SEFR	0.09	0.09	0.00	100.00
DIF	13,044.45	25,574.95	−12,530.50	100.00
DIFR	0.12	0.19	−0.06	100.00
TRF	10,861.05	13,785.40	−2924.36	6.45
TRFR	0.10	0.08	0.02	100.00
WDF	18,485.49	25,529.35	−7043.85	96.77
WDFR	0.17	0.11	0.05	100.00
COF	53,883.81	84,490.74	−30,606.94	3.23
COFR	0.50	0.52	−0.02	100.00
LOS	11.84	18.15	−6.31	25.81

## Data Availability

Data are available upon reasonable request subject to restrictions of patient-privacy protection, institutional policies, and ethical-committee requirements.

## Supplementary Materials

The following supporting information can be downloaded at https://www.mdpi.com/article/10.3390/healthcare14182988/s1, Table S1: Spearman correlation matrix among monthly core indicators; Table S2: COVID-/volume-adjusted sensitivity analysis for core outcomes; Table S3: Surgical-approach-adjusted sensitivity analysis for core outcomes; Figure S1: Monthly trends in total hospitalization costs, consumable fees, and length of stay with DRG implementation and COVID-19-related disturbance windows highlighted.
