# Supplementary material for: Temporal Associations and Heterogeneity of Diagnosis-Related Group (DRG) Payment Reform with Hospitalization Costs Among Patients with Colorectal Cancer in China
Source: Healthcare (Basel). 2026 Sep 12;14(18):2988. doi: 10.3390/healthcare14182988 (PMC13606104; doi:10.3390/healthcare14182988)
Supplement: Supplementary file 1 [file healthcare-14-02988-s001.zip › healthcare-4457498-supplementary.pdf]

Supplementary

Table S1. Spearman correlation matrix among monthly core indicators.

	TF	COF	WDF	DIF	TRF	SEF	LOS
**TF**	1.000	0.907***	0.771***	0.507***	0.583***	0.067	0.491***
**COF**	0.907***	1.000	0.554***	0.351*	0.568***	-0.217	0.259
**WDF**	0.771***	0.554***	1.000	0.248	0.453**	0.203	0.658***
**DIF**	0.507***	0.351*	0.248	1.000	0.053	0.374*	0.374*
**TRF**	0.583***	0.568***	0.453**	0.053	1.000	-0.009	0.141
**SEF**	0.067	-0.217	0.203	0.374*	-0.009	1.000	0.510***
**LOS**	0.491***	0.259	0.658***	0.374*	0.141	0.510***	1.000

Note: Spearman's rho values were calculated using 46 monthly observations. *** P < 0.001, ** P < 0.01, * P < 0.05. TF, total hospitalization cost; COF, consumable fees; WDF, Western medication fees; DIF, diagnostic fees; TRF, treatment fees; SEF, service fees; LOS, length of stay.

Table S2. COVID-/volume-adjusted sensitivity analysis for core outcomes.

**Outcome**	**Model**	**Immediate effect β_2 (SE; 95% CI; P)**	**Trend change β_3 (SE; 95% CI; P)**
Total hospitalization cost (TF, CNY)	Main ITSA	-13,111.73*** (SE=3,250.55; 95% CI -19,741.26 to -6,482.20; P=<0.001)	-1,312.60*** (SE=268.62; 95% CI -1,860.44 to -764.75; P=<0.001)
Total hospitalization cost (TF, CNY)	COVID-/volume-adjusted ITSA	-14,872.51*** (SE=3,117.46; 95% CI -21,258.34 to -8,486.68; P=<0.001)	-1,238.98*** (SE=298.43; 95% CI -1,850.30 to -627.67; P=<0.001)
Consumable fees (COF, CNY)	Main ITSA	-10,322.94*** (SE=1,466.71; 95% CI -13,314.32 to -7,331.57; P=<0.001)	-600.24*** (SE=145.94; 95% CI -897.89 to -302.59; P=<0.001)
Consumable fees (COF, CNY)	COVID-/volume-adjusted ITSA	-10,867.36*** (SE=1,573.58; 95% CI -14,090.70 to -7,644.01; P=<0.001)	-519.81** (SE=155.60; 95% CI -838.54 to -201.08; P=0.002)
Length of stay (LOS, days)	Main ITSA	-0.76 (SE=0.58; 95% CI -1.95 to 0.43; P=0.203)	-0.22*** (SE=0.05; 95% CI -0.32 to -0.12; P=<0.001)
Length of stay (LOS, days)	COVID-/volume-adjusted ITSA	-1.17 (SE=0.70; 95% CI -2.61 to 0.26; P=0.105)	-0.24** (SE=0.07; 95% CI -0.38 to -0.10; P=0.002)

*Note: The COVID-/volume-adjusted model additionally included indicators for April–June 2022 and January–February 2023, as well as monthly admission volume. Newey–West lag order = 4. *** P < 0.001; ** P < 0.01.*

Table S3. Surgical-approach-adjusted sensitivity analysis for core outcomes.

**Outcome**	**Model**	**Immediate effect β_2 (SE; 95% CI; P)**	**Trend change β_3 (SE; 95% CI; P)**
Total hospitalization cost (CNY)	Main ITSA	-13,111.73*** (SE=3,250.55; 95% CI -19,741.26 to -6,482.20; P=<0.001)	-1,312.60*** (SE=268.62; 95% CI -1,860.44 to -764.75; P=<0.001)
Total hospitalization cost (CNY)	Surgical-approach-adjusted ITSA	-13,602.50*** (SE=3,440.04; 95% CI -20,628.00 to -6,576.99; P=<0.001)	-1,184.13*** (SE=295.59; 95% CI -1,787.81 to -580.46; P=<0.001)
Consumable fees (CNY)	Main ITSA	-10,322.94*** (SE=1,466.71; 95% CI -13,314.32 to -7,331.57; P=<0.001)	-600.24*** (SE=145.94; 95% CI -897.89 to -302.59; P=<0.001)

Consumable fees (CNY)	Surgical-approach-adjusted ITSA	-10,868.20*** (SE=1,411.24; 95% CI -13,750.33 to -7,986.06; P=<0.001)	-457.52** (SE=154.85; 95% CI -773.76 to -141.27; P=0.006)
Length of stay (days)	Main ITSA	-0.76 (SE=0.58; 95% CI -1.95 to 0.43; P=0.203)	-0.22*** (SE=0.05; 95% CI -0.32 to -0.12; P=<0.001)
Length of stay (days)	Surgical-approach-adjusted ITSA	-0.85 (SE=0.54; 95% CI -1.96 to 0.25; P=0.126)	-0.20** (SE=0.06; 95% CI -0.31 to -0.08; P=0.002)

*Note: The surgical-approach-adjusted model additionally included the monthly proportion of laparoscopic surgery. Newey-West lag order = 4. *** P < 0.001; ** P < 0.01.*

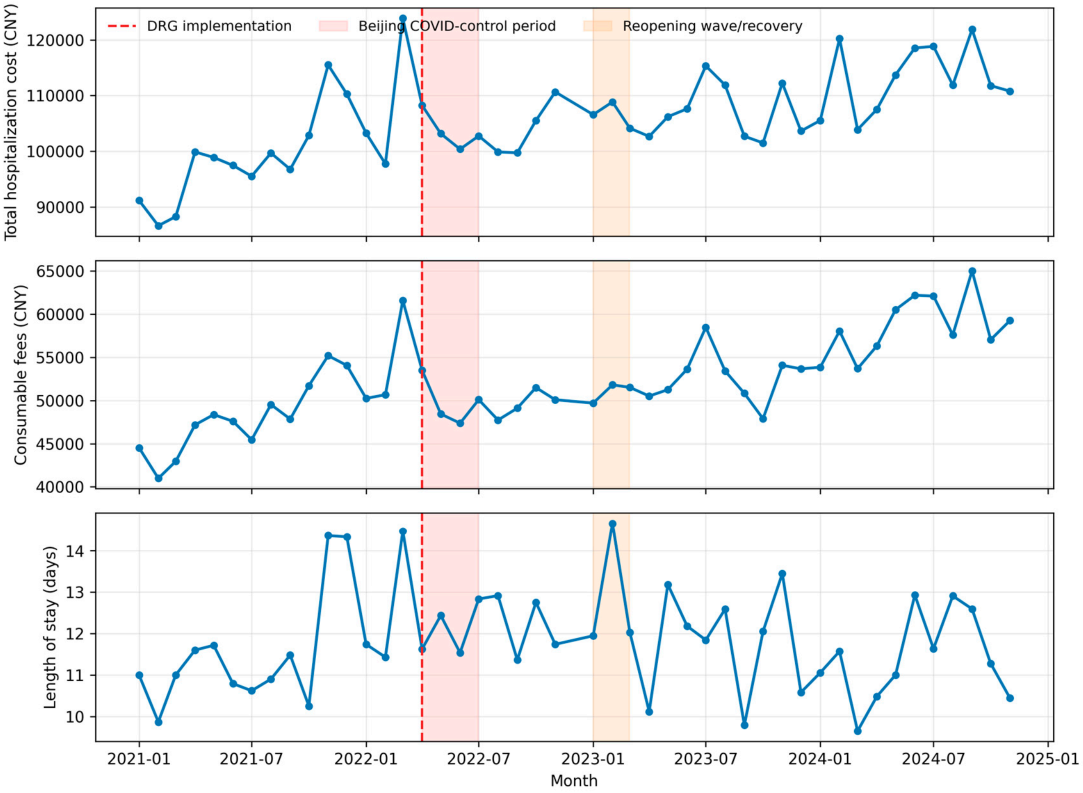

Figure S1. Monthly trends in total hospitalization costs, consumable fees, and length of stay with DRG implementation and COVID-19-related disturbance windows highlighted.
